# Inhibitor of neuronal nitric oxide synthase improves gas exchange in ventilator-induced lung injury after pneumonectomy

**DOI:** 10.1186/1471-2253-12-10

**Published:** 2012-06-21

**Authors:** Evgeny V Suborov, Alexey A Smetkin, Timofey V Kondratiev, Andrey Y Valkov, Vsevolod V Kuzkov, Mikhail Y Kirov, Lars J Bjertnaes

**Affiliations:** 1 Anesthesia and Critical Care Research Group, Institute of Clinical Medicine, Faculty of Health Sciences, University of Tromsø, 9037, Tromsø, Norway; 2 Department of Clinical Pathology, University Hospital of Northern Norway, 9038, Tromsø, Norway; 3 Institute of Medical Biology, University of Tromsø, 9037, Tromsø, Norway; 4 Department of Anesthesiology, Northern State Medical University, Arkhangelsk, Russian Federation

**Keywords:** Pneumonectomy, Mechanical ventilation, nNOS, Pulmonary edema, Sheep, Nitric oxide, Ventilator-induced lung injury, 7-nitroindazole

## Abstract

**Background:**

Mechanical ventilation with high tidal volumes may cause ventilator-induced lung injury (VILI) and enhanced generation of nitric oxide (NO). We demonstrated in sheep that pneumonectomy followed by injurious ventilation promotes pulmonary edema. We wished both to test the hypothesis that neuronal NOS (nNOS), which is distributed in airway epithelial and neuronal tissues, could be involved in the pathogenesis of VILI and we also aimed at investigating the influence of an inhibitor of nNOS on the course of VILI after pneumonectomy.

**Methods:**

Anesthetized sheep underwent right pneumonectomy, mechanical ventilation with tidal volumes (V_T_) of 6 mL/kg and FiO_2_ 0.5, and were subsequently randomized to a protectively ventilated group (PROTV; n = 8) keeping V_T_ and FiO_2_ unchanged, respiratory rate (RR) 25 inflations/min and PEEP 4 cm H_2_O for the following 8 hrs; an injuriously ventilated group with V_T_ of 12 mL/kg, zero end-expiratory pressure, and FiO_2_ and RR unchanged (INJV; n = 8) and a group, which additionally received the inhibitor of nNOS, 7-nitroindazole (NI) 1.0 mg/kg/h intravenously from 2 hours after the commencement of injurious ventilation (INJV + NI; n = 8). We assessed respiratory, hemodynamic and volumetric variables, including both the extravascular lung water index (EVLWI) and the pulmonary vascular permeability index (PVPI). We measured plasma nitrite/nitrate (NOx) levels and examined lung biopsies for lung injury score (LIS).

**Results:**

Both the injuriously ventilated groups demonstrated a 2–3-fold rise in EVLWI and PVPI, with no significant effects of NI. In the INJV group, gas exchange deteriorated in parallel with emerging respiratory acidosis, but administration of NI antagonized the derangement of oxygenation and the respiratory acidosis significantly. NOx displayed no significant changes and NI exerted no significant effect on LIS in the INJV group.

**Conclusion:**

Inhibition of nNOS improved gas exchange, but did not reduce lung water extravasation following injurious ventilation after pneumonectomy in sheep.

## Background

Postpneumonectomy pulmonary edema (PPE) is a subtype of acute lung injury (ALI) developing independently of left ventricular failure, fluid overload or infection, with a prevalence of 2.5–14.3% and a mortality rate of 50–100% [1,2]. By inducing local release of cytokines, the lung injury is not only limited to the airways and the pulmonary vessels, but may progress to circulatory shock and multiple organ dysfunction syndrome (MODS) [3]. Recently, we noticed that pneumonectomy followed by doubling the tidal volume at zero end-expiratory pressure induces ventilator-induced lung injury (VILI) in sheep, which is characterized by derangement of gas exchange, and increments in extravascular lung water and pulmonary vascular permeability [4].

Nitric oxide (NO) generated from L-arginine by calcium-dependent endothelial NO synthase (eNOS) plays an important role in the homeostasis of circulation by modulating vascular tone [5]. Studies performed predominantly on small animals have indicated a role for NO synthesis in the pathogenesis of VILI [5-7]. Recently, investigators observed that eNOS is up-regulated after pneumonectomy in rats [8]. In rabbits, Stromberg and co-workers noticed that mechanical stretch of lung tissue might modulate the NO metabolism [9] and more recent reports revealed that both eNOS and iNOS are up-regulated in VILI in rats and mice [6,7]. Indirect effects of NO include the formation of reactive nitrogen species that damage cells and tissues [10]. Translocation of bacteria through overstretched alveolar epithelium may represent an alternative way of activating inducible NO synthase (iNOS) during excessive ventilation, thereby enhancing the generation of NO [11]. The cytotoxic effect of NO, most likely, increases after combining with highly reactive oxygen species to form peroxynitrite, which supposedly plays an important role in the pathogenesis of MODS [5]. In a previous study, we found that infusion of methylene blue, an unspecific inhibitor of eNOS and iNOS, did not attenuate the emergence of ovine PPE [12]. This motivated us to a search for other inhibitors of NOS that could potentially protect against VILI.

Investigators recently noticed that 7-nitroindazole (NI), an inhibitor of neuronal NOS (nNOS), attenuates ALI after smoke inhalation and burns [13] as well as after inhalation of smoke followed by instillation of live bacteria into the airways of sheep [14]. Although nNOS is abundantly distributed in airway epithelial and neuronal tissues [15], it is still unsettled whether this isoform of NOS is involved in the pathogenesis of VILI after pneumonectomy, and whether NI might antagonize this particular subtype of lung injury. Thus, the aim of the present study was to find out whether NI modulates VILI after pneumonectomy in sheep.

## Methods

The study was approved by the Animal Care Committee of the University of Tromsø under the Norwegian National Animal Research Authority (FOTS id 517/Pro nr 31/07).

### Anesthesia and surgical preparation

Twenty-four yearling sheep 9 ± 5 months of age, weighing 39.5 ± 6.0 kg (mean ± SD) were premedicated with atropine 0.5 mg (Nycomed Pharma AS, Asker, Norway). General anesthesia was induced with an intravenous infusion of thiopental sodium 15–20 mg/kg (Abbott, North Chicago, IL, USA) for endotracheal intubation and maintained with ketamine hydrochloride 4 mg/kg/h (Ketalar, Parke Davis, Solna, Sweden), midazolam 0.4 mg/kg/h (Dormicum®, F. Hoffman-La Roche AG, Basel, Switzerland), pentobarbital 10 mg/kg/h (Pentothal Natrium, NAF, Norway) and fentanyl 12 mcg/kg/h (Hamelin pharma group, Hamelin, Germany) intravenously. Sheep were ventilated with a SERVO*i* respirator (Maquet Critical Care AB, SOLNA, Sweden) in a volume-controlled mode with the following settings: tidal volume (V_T_) 6 mL/kg, positive end-expiratory pressure (PEEP) 4 cm H_2_O, FiO_2_ 0.5, respiratory rate (RR) 25–27 inflations/min and inspiration to expiration ratio (I:E ratio) 1:2. Body temperature was maintained > 38°C by means of heating blankets and a heated, humidified breathing circuit. A 7 F flow-directed pulmonary artery catheter (131HF7; Baxter, Irvine, CA) was advanced into the pulmonary artery through an 8.5 F introducer (CC-350B; Baxter, Irvine, CA) in the left external jugular vein and connected to a pressure transducer (Transpac®III; Abbott, North Chicago, IL). The introducer was also used for infusion of Ringer’s acetate at a rate of 10 ml/kg/h throughout the experiment. Additionally, a 4 F fiberoptic thermistor catheter (PV PV2024L, Pulsion Medical Systems) was placed in the descending thoracic aorta via an introducer in a femoral artery, and a right-sided pneumonectomy was performed, as previously reported [4,12].

### Experimental protocol

After baseline measurements (BL), thoracotomy (TT), and pneumonectomy (PE), the animals were randomly assigned to one of the following experimental groups over 8 hours (h) after PE:

1. Protective ventilation (PROTV; *n* = 8); with V_T_ 6 mL/kg, FiO_2_ 0.5, RR 25–27 inflations/min, I:E ratio 1:2, and PEEP 4 cm H_2_O.

2. Injurious ventilation (INJV; *n* = 8) throughout the post-pneumonectomy period with V_T_ 12 mL/kg, FiO_2_ 0.5, RR 12–13 inflations/min, I:E ratio 1:2, and PEEP 0 cm H_2_O.

3. Injurious ventilation, as above, with subsequent administration of the inhibitor of *n*NOS 7-nitroindazole (N7778, 7-Nitroindazole; Sigma-Aldrich, St. Louis, MO, USA) 1 mg/kg/h dissolved as described by the manufacturer, added to the remainder of Ringer’s acetate and infused intravenously from 2 hours after the commencement of injurious ventilation and throughout the remaining 6 hrs of the experiment (INJV + NI; *n* = 8). The other groups received the solvent dissolved in the Ringer solution only.

### Measurements and samples

Extravascular lung water index (EVLWI), pulmonary vascular permeability index (PVPI), cardiac index (CI), pulmonary blood volume index (PBVI), and global end-diastolic volume index (GEDVI) were assessed by thermo-dye dilution using a COLD-Z021 monitor (Pulsion Medical Systems, Munich, Germany). Pulmonary artery pressure (PAP) and pulmonary artery occlusion pressure (PAOP) were determined with the pulmonary artery catheter, displayed on a 565A Patient Data Monitor (Kone, Espoo, Finland) and stored in Lab View (National Instruments Corporation, Austin, TX, USA). Systemic vascular resistance index (SVRI) and pulmonary vascular resistance index (PVRI) were calculated by the Cold Z-021. All the thermodilution variables were computed as a mean of three measurements, as previously described from our laboratory [4,12].

Total lung and chest quasi static compliance (C_QS_) was calculated as: C_QS_ = V_T_/(P_plat_ – PEEP)/body weight. Blood samples were drawn from the systemic artery - and the pulmonary artery lines and analyzed for arterial (a) and mixed venous ($\bar{v}$) blood gases including PCO_2_, pH, HCO_3_^–^, PO_2_, SO_2_ and hemoglobin concentration (ABL 800 FLEX, Radiometer, Copenhagen, Denmark). Venous admixture was calculated using standard equations [16]. Volumetric and hemodynamic variables, ventilation mechanics and blood gases were determined at baseline (BL), after lateral thoracotomy (TT), after pneumonectomy (PE; time 0), and subsequently at 1 hour intervals until the end of the experiment (in tables, data are presented only at two hour intervals after PE).

### Nitrate/nitrite (NOx) plasma concentrations

The plasma NO concentrations were evaluated by measuring the intermediate and end products, nitrate/nitrite (NOx). Samples were taken at baseline (BL), after pneumonectomy (PE) and at the end of experiment (8 h), and analyzed using a Cayman nitrate/nitrite colorimetric assay kit (Cayman Chemicals, Ann Arbor, MI, USA). Baseline values of NOx were set as 100% and the subsequent values were presented as percentages of the baseline values.

### Lung sampling and histologic examination

The sheep were sacrificed with an intravenous injection of pentobarbital 100 mg/kg (Pentobarbital NAF, Ås Production Lab, Ås, Norway) followed by a bolus injection of 50 mmol KCl (B Braun Melsungen AG, Melsungen, Germany). In five sheep from each group, a histological lung injury score (LIS) was determined by using a modified version of the method proposed by Zhou and colleagues [17], as previously reported from our group [18]. In short, representative tissue blocks from the upper and lower lobes were preserved in 4% formaldehyde, sectioned and stained with haematoxylin and eosin. A pathologist without knowledge of the group identity examined the sections by light microscopy. Each section was scored based on the presence and the degree of edema, neutrophil sequestration, hemorrhage, epithelial desquamation and alveolar hyaline membranes, with zero as the lowest score and four as the highest score. In addition, the percentage of atelectatic alveoli was calculated. Typical photomicrographs were taken using a Leica DM 2500 microscope and a Leica DFC 320 digital camera, with the software Leica IM50 (Leica Microsystems GmbH, Wetzlar, Germany).

### Statistical analysis

For the storage and analysis of the data, we used the SPSS software (SPSS ver. 15, SPSS corp., Illinois, USA). Data were expressed as mean ± SD. The Kolmogorov–Smirnov test was used to assess the data distribution. Normally distributed data were assessed by two-way analysis of variance (ANOVA). If the F value was statistically significant, ANOVA was followed by Scheffe’s *post hoc* test and test of contrasts for intergroup and intragroup comparisons, respectively. If a normal distribution could not be demonstrated, such as for the lung injury score, the Kruskal–Wallis test was used to detect differences between the groups. P < 0.05 was regarded as statistically significant.

## Results

### Volumetric and hemodynamic changes

**Figure 1:**
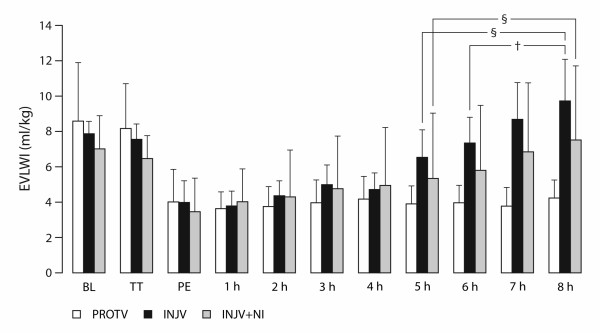
**Extravascular lung water index (EVLWI) in anesthetized sheep.** Baseline (BL), after right-sided thoracotomy and pneumonectomy (PE = time 0 hour) subsequently followed by injurious ventilation. 2 h – 8 h are time points in hours after PE. 7-nitroindazole (NI) was administered from time 2 h and throughout. Protectively ventilated group (PROTV; n = 8), injuriously ventilated group (INJV; n = 8), injuriously ventilated group treated with intravenously infused 7-nitroindazole (INJV + NI; n = 8). Data are presented as the mean ± standard deviation. ^§^ P < 0.05 within group in comparison with PE; ^†^ P < 0.05 between PROTV and INJV group.

All the sheep survived without signs of aspiration, barotrauma or severe blood loss. We found no significant volumetric or hemodynamic differences between the groups at BL or PE. As shown in Figure 1, after PE, EVLWI increased by 88% and 177% in the INJV + NI group and the INJV group, respectively (P < 0.05). Correspondingly, PVPI increased by 100% and 250% (P < 0.05), respectively (Table1). The only intergroup differences in EVLWI and PVPI were found between the INJV group and the PROTV group (P < 0.05; Figure 1 and Table1).

**Table 1:** Hemodynamic and volumetric variables in sheep subjected to pneumonectomy and injurious ventilation

**Variable**	**Group**	**BL**	**PE**	**2 h**	**4 h**	**6 h**	**8 h**
**PVPI**	PROTV	0.03 ± 0.01	0.02 ± 0.00	0.02 ± 0.00	0.02 ± 0.01	0.02 ± 0.01	0.02 ± 0.01^†^
	INJV	0.04 ± 0.01	0.02 ± 0.01	0.03 ± 0.01	0.02 ± 0.01	0.04 ± 0.02	0.07 ± 0.03^§^
	INJV + NI	0.04 ± 0.02	0.03 ± 0.02	0.03 ± 0.03	0.03 ± 0.03	0.04 ± 0.05^§^	0.06 ± 0.05^§^
**PBVI (mL/m**^**2**^**)**	PROTV	285 ± 127	196 ± 74	210 ± 75	215 ± 62	206 ± 69	210 ± 65
	INJV	229 ± 38	183 ± 49	179 ± 54	208 ± 31	181 ± 41	162 ± 47
	INJV + NI	208 ± 42	163 ± 55	171 ± 30	173 ± 25	181 ± 47	162 ± 38
**GEDVI**	PROTV	602 ± 127	557 ± 175	537 ± 138	539 ± 144	567 ± 124	612 ± 144
**(mL/m**^**2**^**)**	INJV	603 ± 72	532 ± 70	527 ± 78	573 ± 65	598 ± 111^§^	622 ± 160^§^
	INJV + NI	615 ± 80	540 ± 48	594 ± 118	553 ± 66	617 ± 50^§^	590 ± 65^§^
**CI**	PROTV	3.4 ± 0.8	3.9 ± 1.9	4.5 ± 1.7	4.4 ± 1.7	4.7 ± 1.4	4.6 ± 1.6
**(L/min/m**^**2**^**)**	INJV	3.6 ± 0.5	3.7 ± 0.7	4.4 ± 1.1	5.2 ± 1.2^§^	5.2 ± 1.3^§^	4.9 ± 1.7
	INJV + NI	4.1 ± 0.7	2.9 ± 0.5	3.8 ± 0.6^§^	3.7 ± 0.6^§^	3.9 ± 0.6^§^	3.5 ± 0.6
**PAP (mm Hg)**	PROTV	12 ± 2	19 ± 5	20 ± 3	21 ± 4	20 ± 4	22 ± 7
	INJV	12 ± 3	18 ± 5	21 ± 4^§^	25 ± 5^§^	28 ± 6^§,†^	31 ± 6^§,†^
	INJV + NI	12 ± 2	17 ± 2	20 ± 4^§^	23 ± 7^§^	26 ± 6^§^	28 ± 6^§^
**PAOP (mm Hg)**	PROTV	6 ± 3	6 ± 3	6 ± 3	9 ± 4^§^	9 ± 4^§^	8 ± 4^§^
	INJV	5 ± 2	8 ± 3	8 ± 3	12 ± 4^§^	14 ± 5^§,†^	16 ± 4^§,†,#^
	INJV + NI	6 ± 2	8 ± 3	10 ± 3^§^	9 ± 4	11 ± 4^§^	10 ± 3^§^
**SVRI (dyn sec/cm**^**-5**^**/m**^**2**^**)**	PROTV	2096 ± 641	2757 ± 878	2001 ± 749^§^	2039 ± 923^§^	1851 ± 877^§^	1889 ± 872^§^
	INJV	2278 ± 418	2389 ± 572	2021 ± 592	1682 ± 491^§^	1547 ± 480^§^	1717 ± 514^§^
	INJV + NI	2034 ± 371	2743 ± 571	2352 ± 647	2198 ± 562	2299 ± 432	2332 ± 538
**PVRI (dyn sec/cm**^**-5**^**/m**^**2**^**)**	PROTV	129 ± 53	286 ± 104	258 ± 123	247 ± 146	220 ± 81^§^	253 ± 136
	INJV	164 ± 73	231 ± 34	238 ± 54	212 ± 51	233 ± 78	276 ± 125
	INJV + NI	128 ± 56	266 ± 103	242 ± 72	299 ± 130	333 ± 155	426 ± 122^‡^

Pulmonary vascular permeability index (PVPI), cardiac index (CI), pulmonary blood volume index (PBVI), global end-diastolic volume index (GEDVI), pulmonary artery pressure (PAP), pulmonary artery occlusion pressure (PAOP), systemic vascular resistance index (SVRI) pulmonary vascular resistance index (PVRI). Baseline (BL), thoracotomy (TT), pneumonectomy (PE), 2 h – 8 h are time points in hours after PE. Data are presented as the mean ± standard deviation. Protectively ventilated group (PROTV; n = 8), injuriously ventilated group (INJV; n = 8), injuriously ventilated group treated with intravenously infused 7-nitroindazole (INJV + NI; n = 8). ^§^ P < 0.05 within group in comparison with PE; ^†^ P < 0.05 between PROTV and INJV groups; ^‡^ P < 0.05 between INJV + NI and PROTV groups; ^#^ P < 0.05 between INJV + NI and INJV groups.

As depicted in Table1, PBVI displayed no significant changes whereas GEDVI and CI increased transiently in both the injuriously ventilated groups (P < 0.05). PAP and PAOP also increased in the injuriously ventilated groups, as compared to PE and to the PROTV group (P < 0.05). At cessation of the experiments, PAOP decreased in NI-treated sheep as compared to non-treated injuriously ventilated animals (P < 0.05). In the PROTV and the INJV groups, SVRI declined after PE (P < 0.05), but NI prevented this decrease and increased PVRI, as compared to the PROTV group (P < 0.05) (Table1).

### Gas exchange and ventilation mechanics

**Figure 2:**
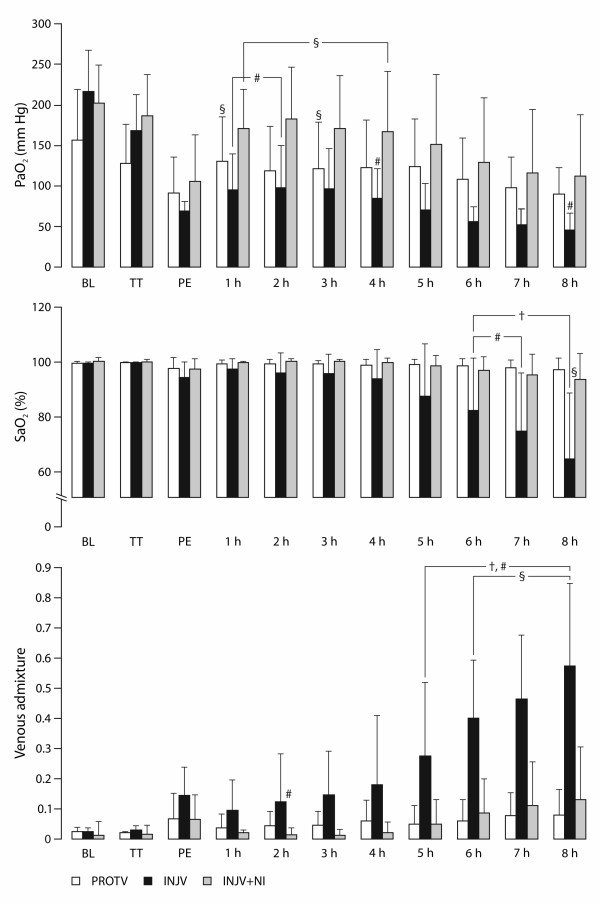
**Arterial oxygen partial pressure (PaO**_**2**_**), saturation (SaO**_**2**_**) and venous admixture in anesthetized sheep.** Baseline (BL), after right-sided thoracotomy (TT) and after pneumonectomy (PE = time, 0 hour) subsequently followed by injurious ventilation. 2 h – 8 h are time points in hours after PE. 7-nitroindazole (NI) was administered from time 2 h and throughout. Protectively ventilated group (PROTV; n = 8), injuriously ventilated group (INJV; n = 8), injuriously ventilated group treated with intravenously infused 7-nitroindazole (INJV + NI; n = 8). Data presented as the mean ± standard deviation. ^§^ P < 0.05 within group in comparison with PE; ^†^ P < 0.05 between PROTV and INJV, ^#^ P < 0.05 between INJV and INJV + NI groups.

Blood gases displayed no significant intergroup differences at BL or at PE. As depicted in Figure 2, PaO_2_ and SaO_2_ increased towards the end of experiment in the INJV + NI group (P < 0.05) and venous admixture increased in the INJV group (P < 0.05). PaCO_2_ increased and pH decreased in the INJV group, in comparison with PE and with the other groups (P < 0.05; Table2). After PE, SvO_2_ increased transiently in all the sheep, and in the INJV group Hb increased in comparison with both intragroup PE and with the other groups (P < 0.05; Table2).

**Table 2:** Ventilation, blood gases and metabolic parameters in sheep subjected to pneumonectomy and injurious ventilation

**Variable**	**Group**	**BL**	**PE**	**2 h**	**4 h**	**6 h**	**8 h**
**PaCO**_**2**_**(mm Hg)**	PROTV	41.9 ± 5.3	34.5 ± 2.9	37.2 ± 4.2	36.4 ± 3.5	37.4 ± 3.9	38.6 ± 6.0
	INJV	44.2 ± 3.4	42.1 ± 7.1^†^	39.2 ± 6.7	45.1 ± 20.9	55.1 ± 24.9	65.8 ± 30.4^§,†,#^
	INJV + NI	40.6 ± 3.2	36.2 ± 4.4	32.5 ± 5.2	35.8 ± 4.2	39.8 ± 6.7	37.1 ± 3.8
**pH**	PROTV	7.44 ± 0.05	7.50 ± 0.05	7.49 ± 0.05	7.49 ± 0.06	7.48 ± 0.07	7.45 ± 0.11
	INJV	7.44 ± 0.05	7.44 ± 0.07	7.45 ± 0.08	7.42 ± 0.13	7.35 ± 0.13^§,†^	7.28 ± 0.17^§,†,#^
	INJV + NI	7.43 ± 0.03	7.49 ± 0.06	7.51 ± 0.05	7.46 ± 0.05	7.44 ± 0.07	7.45 ± 0.06
**SvO**_**2**_**(%)**	PROTV	71 ± 9	58 ± 13	72 ± 11^§^	70 ± 9^§^	68 ± 6	62 ± 11
	INJV	78 ± 4	62 ± 9	73 ± 9^§^	74 ± 10^§^	62 ± 22	46 ± 25
	INJV + NI	77 ± 7	55 ± 6	72 ± 14^§^	74 ± 12^§^	70 ± 10^§^	59 ± 16
**Hb (g/L)**	PROTV	87 ± 6	87 ± 5	94 ± 13	92 ± 14	91 ± 13	85 ± 12
	INJV	88 ± 5	92 ± 6	103 ± 11^§^	109 ± 13^§,†^	115 ± 10^§,†,#^	124 ± 15^§,†,#^
	INJV + NI	81 ± 5	84 ± 9	89 ± 7	92 ± 8	94 ± 11	96 ± 10
**Ppeak**	PROTV	20 ± 7	42 ± 9	40 ± 9^†,‡^	45 ± 16^‡^	47 ± 15^†,‡^	50 ± 18^†,‡^
**(cmH**_**2**_**O)**	INJV	17 ± 3	36 ± 4	46 ± 6^§^	49 ± 5^§^	57 ± 11^§^	61 ± 16^§^
	INJV + NI	20 ± 4	43 ± 9	55 ± 12^§^	60 ± 15^§^	68 ± 18^§^	67 ± 18^§^
**C**_**QS**_	PROTV	0.53 ± 0.20	0.25 ± 0.08	0.27 ± 0.07^§^	0.26 ± 0.07	0.25 ± 0.06	0.24 ± 0.05
**(mL/cmH**_**2**_**O/kg)**	INJV	0.45 ± 0.05	0.21 ± 0.03	0.32 ± 0.04^§^	0.30 ± 0.05^§^	0.25 ± 0.05^§^	0.23 ± 0.06
	INJV + NI	0.40 ± 0.09	0.26 ± 0.07	0.35 ± 0.08	0.33 ± 0.10	0.28 ± 0.07	0.27 ± 0.08

Partial pressure of carbon dioxide in arterial blood (PaCO_2_), mixed venous oxygen saturation (SvO_2_), hemoglobin concentration (Hb), peak airway pressure (Ppeak), quasi static compliance (C_QS_). Baseline (BL), pneumonectomy (PE), 2 h – 8 h are time points in hours after PE. Data are presented as the mean ± standard deviation. Protectively ventilated group (PROTV; n = 8), injuriously ventilated group (INJV; n = 8), injuriously ventilated group treated with intravenously infused 7-nitroindazole (INJV + NI; n = 8). ^§^ P < 0.05 within group in comparison with the 0 hours; ^†^ P < 0.05 between PROTV and INJV groups; ^‡^ P < 0.05 between INJV + NI and PROTV groups; ^#^ P < 0.05 between INJV + NI and INJV groups.

We found no intergroup differences in ventilation mechanics at baseline. In the injuriously ventilated sheep, Ppeak increased in parallel with a decrease in C_QS_ (Table2). The changes in Ppeak had similar patterns in both injuriously ventilated groups and differed both from their respective intragroup values at PE and from the PROTV group (P < 0.05). Although not statistically significant, C_QS_ tended to be higher in NI-treated animals (Table2).

### NOx

**Figure 3:**
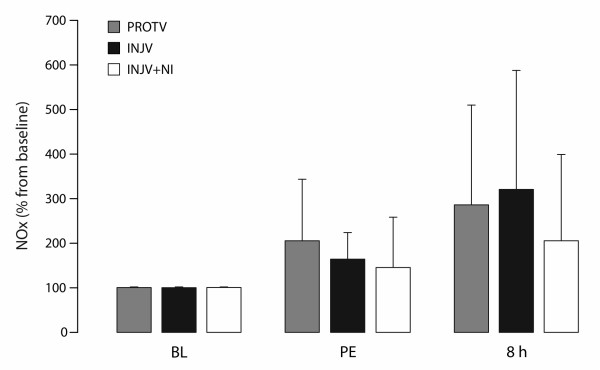
**Changes in plasma concentrations of NOx in anesthetized sheep.** Baseline (BL), after pneumonectomy (PE) subsequently followed by injurious ventilation and after euthanasia at 8 hours (8 h). 7-nitroindazole (NI) was administered from time 2 h (hours) and throughout. Protectively ventilated group (PROTV; n = 8), injuriously ventilated group (INJV; n = 8), injuriously ventilated group treated with intravenously infused 7-nitroindazole (INJV + NI; n = 8). Baseline values of NOx were set as 100% and the subsequent values were calculated as percentages of the baseline values. Data are presented as the mean ± standard deviation.

The plasma concentration of NOx tended to increase throughout the experiment, but with no significant differences within – or between the groups (Figure 3).

### Lung injury score

**Figure 4:**
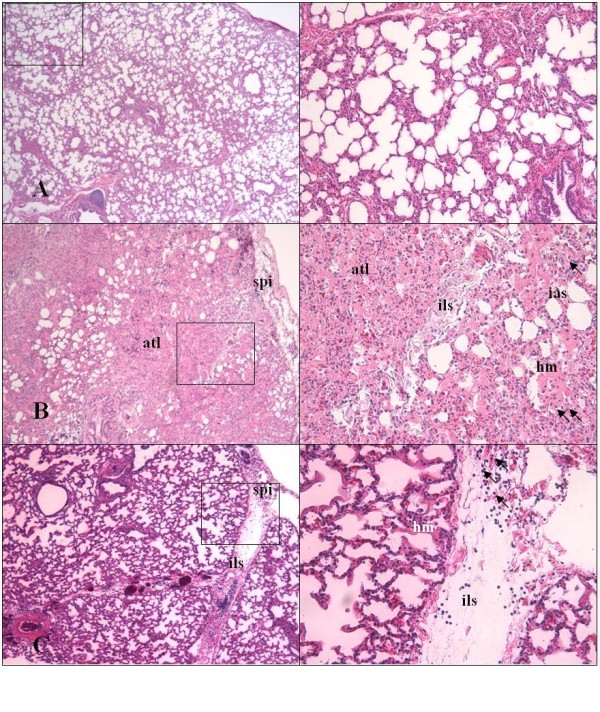
**Lung histology.** Photomicrographs of hematoxylin - and eosin-stained left lung lower lobe specimens from sheep subjected to right pneumonectomy followed by an 8 h period of one-lung ventilation. Magnification x 25 (left). Areas within the squares are magnified x 100 (right). **Panel A**: protective ventilation (PROTV group) showing no pathologic changes. **Panel B**: injurious ventilation (INJV group). **Panel C**: injurious ventilation followed by intravenous infusion of 7-nitroindazole (INJV + NI group). Atelectasis (atl), interstitial edema with thickening of interalveolar - and interlobular septa (ias and ils), thickening of the subpleural interstitium (spi), extravasation of neutrophils (arrows), formation of hyaline membranes (hm).

Total lung injury score (LIS) was significantly higher in the INJV + NI group in comparison with the PROTV group (P < 0.05) (Table3). As the individual data are concerned, hyaline membrane formation was more extensive in the INJV group as compared to the PROTV group (P < 0.05) and significantly more neutrophils were found in the NI-treated - as compared to the non-treated INJV group (P < 0.05). We found a four times higher percentage of atelectasis in the INJV group in comparison with the NI-treated group (28% vs. 7%; not significant). Figure 4 shows photomicrographs of typical lung sections with low power fields (left). The high power fields (right) are displayed by the marked squares. In the PROTV group (A), we found no pathological changes. The INJV group (B) displays hyperemia accompanied by extensive atelectasis (atl), interstitial edematous thickening of the interalveolar - and interlobular septa (ias and ils) - and of the subpleural interstitium (spi), and hyaline membrane (hm) formation, and extravasation of neutrophils (arrows). The INJV + NI group (C), displays nearly the same changes with less atelectasis (C).

**Table 3:** Histological lung injury score (LIS) of sheep subjected to pneumonectomy and injurious ventilation

**Group**	**Edema**	**Neutrophil infiltration**	**Hemorrhage**	**Hyaline membranes**	**Epithelial desquamation**	**Σ**
**PROTV**	1.0 (0.0 to 1.0)	1.0 (0.0 to 2.0)	1.0 (0.0 to 3.0)	0.0 (0.0 to 2.0)	0.0 (0.0 to 1.0)	4.0 (1.0 to 7.0)
**INJV**	1.0 (1.0 to 3.0)	1.0 (0.0 to 3.0)	1.0 (0.0 to 2.0)	2.0 (2.0 to 3.0)^†^	1.0 (0.0 to 2.0)	5.0 (4.0 to 12.0)
**INJV + NI**	2.5 (2.0 to 3.0)‡	3.0 (2.0 to 3.0)‡^,#^	2.5 (1.0 to 3.0)	1.0 (1.0 to 2.0)‡	2.5 (2.0 to 3.0)	11.0 (11.0 to 12.0)‡

Data presented as the median (minimum to maximum). Protectively ventilated group (PROTV; n = 5), injuriously ventilated group (INJV; n = 5), injuriously ventilated group treated with intravenously infused 7-nitroindazole (INJV + NI; n = 5).

‡ P < 0.05 between INJV + NI and PROTV; ^#^ P < 0.05 between INJV + NI and INJV; ^†^ P < 0.05 between PROTV and INJV group.

## Discussion

The present study confirms our previous findings in sheep: pneumonectomy followed by one-lung ventilation with excessive tidal volumes and zero end-expiratory pressure promotes lung injury, as characterized by increased pulmonary vascular pressure and permeability, and accumulation of extravascular lung water in concert with derangements of gas exchange [4,12]. Continuous infusion of the inhibitor of nNOS, 7-nitroindazole (NI), from two hours after the start of injurious ventilation dampened the decrease in oxygenation and the respiratory acidosis. Apparently, NI also delays the emerging increments in extravascular lung water and pulmonary vascular permeability, although without reaching significant intergroup differences.

The absence of changes in PBVI in the early postpneumonectomy period confirmed our previous finding that ovine ventilator-induced lung injury after pneumonectomy is not a result of cardiac failure or fluid volume overload [4,12]. This notion is also supported by the observation of no significant differences in GEDVI, which is another marker of preload, between injuriously and protectively ventilated animals [19]. We speculate that the accumulation of EVLW could be caused by a combination of increased pulmonary microvascular pressure and permeability, as evidenced by the elevations of PAP, PAOP and PVPI in the injuriously ventilated groups. A decrease in vascular capacity after volume reduction surgery in the presence of transiently increased cardiac output could, at least, partly explain the rises in PAP and PAOP. However, despite the fact that no significant intergroup differences were noticed, the latter variables tended to be lower in NI-treated sheep (Table1). These findings are consistent with previous observations from our own group as well as from investigators employing a porcine model of pneumonectomy, who found that mean PAP increased significantly immediately after the lung was removed [4,20]. Stable PAP and PAOP without any signs of lung edema in protectively ventilated sheep confirm the findings of previous workers, who were unable to provoke postpneumonectomy pulmonary edema in dogs if the left heart filling pressures were kept within normal ranges [21].

We interpret the increase in PVPI as the result of an inflammatory response to the combination of pneumonectomy and injurious ventilation. Other workers have noticed that pneumonectomy might predispose to thrombus formation and embolization of the contralateral pulmonary artery [22]. The increased pro-coagulant activity could therefore be suspected of stimulating both the coagulation and the inflammation in the forefront of the physical stress that was induced by the injurious ventilation [23,24]. The stimulation of inflammation is supported by the observation that LIS is higher in the injuriously ventilated group, as compared to the PROTV group (Table3). Surprisingly, neutrophil infiltration was higher in the NI-treated sheep- as the only factor differing significantly between the injuriously ventilated animals. A literature search revealed no previous studies focusing on inflammation and lung histological changes after combined pneumonectomy and injurious ventilation in sheep. Our findings contrast with those reported from ovine studies of lung injury after smoke inhalation followed by bronchial instillation of live bacteria or burns, both showing improved lung histology and reduced inflammation after NI [14,25]. We have no good explanation of these discrepancies except for the fact that LIS showed great variations and the sample size consisted of only five sheep in each group. The assumption that coagulation and inflammation might have acted together to provoke lung injury in these animals is also consistent with recent studies demonstrating that injurious ventilation alone can lead to a biotrauma, which might increase the pulmonary microvascular permeability [26]. Worthy of comment in support of the contention that inflammation is involved, is also the observation in mice that Toll-like receptor 4, which activates the innate immune system by responding to lipopolysaccharide from Gram-negative bacteria, also initiates the innate immune response to ventilator-induced lung injury [27,28]. However, it remains to be settled if and to what extent these observations can be of any relevance to the emergence of VILI in large animals.

The increase in EVLWI is consistent with the findings of investigators who noticed a positive correlation between the degree of lung inflation and microvascular pore radius in mechanically ventilated sheep. At high inflation pressure and volume, the restriction of solute diffusion was lost resulting in a net movement of liquid into the alveoli. The authors suggested that as the lung epithelium is progressively stretched there is an opening up of water-filled channels between the alveolar cells resulting in increased extravascular water accumulation [29]. In addition to the increased pulmonary fluid filtration, mechanical ventilation has been shown to contribute to the increased EVLWI by impeding the clearance of lung lymph in anesthetized dogs and sheep [30,31].

In the present study, Hb concentration was significantly lower in the NI-treated – as compared to the non-treated injuriously ventilated animals (Table2). Because EVLWI did not differ significantly between the injuriously ventilated groups, it is close at mind to believe that the fluid leak prompting the increase in Hb concentration, which was actually antagonized by NI, possibly could have occurred in extrapulmonary parts of the circulation. However, the mechanism by which NI could have acted to increase the microvascular reabsorption, or lymphatic clearance from other parts of the circulation, remains mere speculation and cannot be further elucidated by this study.

Protective ventilation of the remaining lung after PE together with increased FiO_2_ was sufficient to maintain normal gas exchange, as previously reported from our group [12]. Although we were unable to demonstrate significant changes in the plasma concentrations of NOx (Figure 3), we speculate that enhanced local production of NO by eNOS [8] might have contributed to vasodilatation and loss of hypoxic pulmonary vasoconstriction (HPV) in atelectatic and poorly ventilated lung areas (Table3), thereby causing a decrease in arterial oxygenation. Evidently, these non-treated animals had more extensive ventilation/perfusion (V/Q) disturbances, including PaO_2_/FiO_2_ ratio reaching ARDS criteria, increased venous admixture, and higher alveolar dead-space, as indicated by the rise in PaCO_2_. Albeit not significantly different, there was a trend towards lower C_QS_ and histologically more atelectases in the INJV group. In contrast, NI-treated animals displayed improvements of the deranged gas exchange (Figure 2, Table2) and less severe atelectasis. We believe that the gas exchange improved as a result of more favorable V/Q distribution due to the combined effects of reinforcement of HPV, less atelectasis and slightly increased C_QS_ after administration of NI, although none of the latter changes were significant. However, whether the possible reinforcement of HPV was caused by a specific inhibitory effect on nNOS or by unspecific effects on eNOS or iNOS, cannot be settled from these experiments [25]. Similar findings (i.e. attenuation of pulmonary dysfunction) have been described by our group and others by using inhibitors of various isoforms of NOS on different ovine models of acute lung injury [25,32-34].

Since postpneumonectomy pulmonary edema occurred only in sheep subjected to injurious ventilation and not in protectively ventilated animals, we assume that ventilation with excessive tidal volumes and zero end-expiratory pressure might have played a pivotal role for this complication to develop. Consistently, we also found the most severe interstitial edema together with neutrophil infiltration and hyaline membrane formation in the injuriously ventilated groups, as confirmed by LIS (Table3, Figure 4). Our findings agree with those of clinical investigators, who described increased pulmonary microvascular permeability, loss of endothelial integrity and alveolar edema after lung resection, as well as neutrophil activation after esophagectomy and pneumonectomy [35,36]. In contrast, investigators studying ALI in sheep after smoke inhalation in combination with airway instillation of bacteria - or with third degree burns, noticed that inhibition of nNOS both reduced the airway obstruction and improved the ventilation mechanics and the gas exchange [13,14,32]. Recently, the latter investigators noticed that ovine sepsis is associated with early and transient rises in the expression of eNOS and iNOS, while expression of nNOS remains unchanged [37]. Whether corresponding changes occur in response to excessive one-lung ventilation after pneumonectomy has not been settled.

The present study has several limitations: we provide no evidence of increased production of NO (Figure 3), and due to technical reasons, we were not able to determine the expressions of *n*NOS, eNOS and iNOS, or their proteins in lung tissue. However, higher vascular tones both in the systemic - and the pulmonary circulations, and less venous admixture in NI-treated sheep (Table1, Figure 2), suggest that at least, a partial inhibition of NOS has taken place, most likely of eNOS [8]. We also admit the lack of a group demonstrating the effects of NI alone and a lack of dose–response data, butother investigators registered no changes in hemodynamics or gas exchange that could be attributed to the use of NI per se [32]. These investigators also found that a dose of NI 1 mg/kg/h was sufficient to reduce NOx plasma levels and to preserve HPV, whereas no further increase in HPV was obtained with higher doses [25]. These investigators started administration of NI one hour after the commencement of the injurious stimulation on awake sheep over 24–48 hrs [13,14,25]. In our study, NI was administered from two hours after the start of injurious ventilation, which lasted only 8 hrs after the pneumonectomy. It is therefore likely that the significant effect on gas exchange and the tendency towards a significantly increased difference in EVLWI between the injuriously ventilated groups had continued beyond 8 hrs. Investigators studying the effect of NI on ovine lung injury after smoke inhalation and burn, reported the first sign of improved oxygenation between 6 and 12 hours after the injury, as assessed by a decreased intrapulmonary shunt [32]. In contrast to our first study employing the same animal model, some of the present data displayed a greater variability, which is caused, at least in part, by the fact that muscle relaxants were not used [4]. We also notice as a shortage the sample sizes of only five animals in each group examined for LIS.

## Conclusion

Pneumonectomy followed by one-lung ventilation using high tidal volumes and zero end-expiratory pressure induced a lung injury in sheep, which is characterized by pulmonary edema and derangement of gas exchange. Intravenous infusion of the inhibitor of *n*NOS, 7-nitroindazole, started two hours after the pneumonectomy and the start of injurious ventilation, improved gas exchange but did not prevent lung injury with pulmonary edema in these experiments over eight hours. Further experiments of longer duration seem to be necessary to find out whether 7-nitroindazole alleviates this subtype of ventilator-induced lung injury, as experienced in other ovine models of acute lung injury.

### Key messages

· Pneumonectomy followed by injurious one-lung ventilation results in increments in pulmonary vascular permeability and pulmonary edema in concert with derangement of gas exchange.

· Treatment with the inhibitor of *n*NOS, 7-nitroindazole, improves gas exchange, but does not counteract pulmonary edema and morphological injury to the pulmonary parenchyma in this model of acute lung injury.

## Abbreviations

ALI, Acute lung injury; ANOVA, Two-way analysis of variance; BL, Baseline; CI, Cardiac index; CQS, Lung quasi static compliance; EVLWI, Extravascular lung water index; GEDVI, Global end-diastolic volume index; HPV, Hypoxic pulmonary vasoconstriction; I:E, Inspiration to expiration ratio; INJV, Injuriously ventilated group; iNOS, Inducible NO synthase; IV, Intravenously; LIS, Lung injury score; MODS, Multiple organ dysfunction syndrome; MV, Mechanical ventilation; NI, 7-nitroindazole; nNOS, Neuronal NO synthase; NO, Nitric oxide; NOx, Nitrate/nitrite; PAOP, Pulmonary artery occlusion pressure; PAP, Mean pulmonary artery pressure; PBVI, Pulmonary blood volume index; PE, Pneumonectomy; PEEP, Positive end-expiratory pressure; PPE, Postpneumonectomy pulmonary edema; Ppeak, Peak airway pressure; Pplateau, Plateau pressure; PROTV, Protectively ventilated group; PVPI, Pulmonary vascular permeability index; PVRI, Pulmonary vascular resistance index; RAP, Mean right atrial pressure; RR, Respiratory rate; SVRI, Systemic vascular resistance index; TT, Thoracotomy; V/Q, Ventilation/perfusion; VILI, Ventilator-induced lung injury; VT, Tidal volume.

## Competing interests

None of the authors have declared any competing interests for this study.

## Authors’ contributions

**EVS** performed the experiments, analyzed the data and drafted the manuscript. **TVK** assisted technically under the instrumentation and experimentation. **AAS** assisted technically under the instrumentation and experimentation and drew the figures. **VVK** drew figures, analyzed the data and contributed to the discussion of the results. **AYV** performed the histological analysis of the samples and lung injury score calculations and drafted parts of the manuscript dealing with morphology. **LJB** and **MYK** participated in the administration and design of the study, and drafted the manuscript. All authors have read and approved the final manuscript.

## Pre-publication history

The pre-publication history for this paper can be accessed here:

http://www.biomedcentral.com/1471-2253/12/10/prepub
